# Specific Brain Activity During Theory of Mind Tasks in Autistic Individuals: A Meta‐Analysis of fMRI Studies

**DOI:** 10.1002/pchj.70060

**Published:** 2026-01-07

**Authors:** Hanran Li, Yihui Wang, Lei Chang, Li Yi, Lai Na Siu, Juan Zhang

**Affiliations:** ^1^ Centre for Cognitive and Brain Sciences University of Macau Macau China; ^2^ Department of Psychology, Faculty of Social Science University of Macau Macau China; ^3^ Faculty of Education University of Macau Macau China; ^4^ School of Psychological and Cognitive Sciences & Key Laboratory of Behavior and Mental Health Peking University Beijing China; ^5^ IDG/McGovern Institute for Brain Research at PKU, Peking University Beijing China

**Keywords:** activation network mapping, autism, fMRI, meta‐analysis, theory of mind

## Abstract

Autistic individuals exhibit differences in Theory of Mind (ToM) compared to neurotypical (NT) individuals. The aim of this study was to meta‐analyse the neural correlates that contributed to the manifestation of the expression differences in ToM between autistic individuals and the NT population. A total of 328 autistic participants and 314 NT participants from 18 studies were included. We adopted Activation Network Mapping, which is a novel neuroimaging meta‐analysis method based on activation seeds and functional connectivity to identify brain networks, to investigate how the ToM network of the autistic group differed from that of the NT group. The thalamus and precuneus robustly participated in the ToM network of the autistic group. Moreover, the temporoparietal junction and the right hemisphere of the limbic system, especially the thalamus, caudate, and cingulum, were less involved in the autistic group's ToM network, compared to the NT group. Our findings provide the first quantitative evidence supportive of the distinct patterns in the ToM brain network in the autistic population. The current findings indicate that the primary difference in ToM task performance in autistic individuals may stem from altered information processing mechanisms rather than deficits in core ToM abilities.

## 1 Introduction

Autism spectrum disorder (ASD) is a neurodevelopmental condition and its core characterisations are persistent communication and social interaction difficulties, repetitive behaviour and limited interests according to *DSM‐5* (American Psychiatric Association 2013). These characterisations impede the autistic population's social engagement and lead to different behavioural patterns. Divergent accounts have been proposed to explain the challenges in autistic individuals. One influential account from Baron‐Cohen et al. (1985) argues that the varying social behaviours of the autistic population are caused by the lack of Theory of Mind (ToM) ability which is defined as the capacity to understand the thoughts and feelings of others, because ToM is important for administering actions and making predictions (Ho et al. 2022).

ToM, a high‐order cognitive skill, consists of diverse basic component processes. A potential comprehensive framework suggests that environmental perception, information processing and belief or intention formation constitute the flow of ToM (Schaafsma et al. 2015; Schurz et al. 2021; Stacy et al. 2024; Yang et al. 2015). Numerous studies have demonstrated that the behavioural performance of ToM in the autistic population differs from that in the neurotypical (NT) population (Dubey et al. 2015; Shic et al. 2014; Yang et al. 2017). We will next outline existing research related to the behavioural comparisons between the autistic and NT populations on the basic component processes mentioned above.

The perception of the environment is generally defined as apprehending the features of the physical environment through sensory input. Autistic individuals often demonstrate different perceptions of the environment from the NT population. Numerous studies have been conducted with visual stimuli because approximately 80% of the information is visual (Haupt and Huber 2008). There are three major findings from existing literature. Firstly, autistic children demonstrate enhanced ability in visual search tasks because they seem to be more inclined to take care of detailed information (Joseph et al. 2009; O'Riordan et al. 2001; Shah and Frith 1983). This feature of environmental perception remains in autistic adulthood (Constable et al. 2010). Secondly, in response to motion stimuli, autistic individuals pay more attention to repetitive movements, such as a rolling wheel, as compared to irregular motion (Gong et al. 2021). There is a deficit in the perception of biological motion in the autistic population (Federici et al. 2020; Klin et al. 2009). Lastly, faces are a crucial part of the environmental information that humans process daily. Autistic individuals tend to show less interest in faces than objects without social information (Vacas et al. 2021). The autistic population also exhibits diminished interest in gaze fixation (Dalton et al. 2005).

The information processing can be interpreted as the decoding or deconstruction of information in human brains, including storing, recognising, extracting features, sorting and other complex processing (Schaafsma et al. 2015). Working memory is a typical example of the storing function in information processing. Meta‐analysis studies on working memory in autistic individuals reveal poor abilities in both spatial and verbal working memory (Demetriou et al. 2018; Wang et al. 2017). In the meantime, autistic individuals often exhibit reduced accuracy and slower response in facial identity recognition tasks compared to NT individuals (Stantic et al. 2022; Weigelt et al. 2012). Emotional classification is a widely used paradigm to investigate the ability to extract features and sorting. Adopting this task, previous researchers reveal that autistic individuals struggle to classify emotions accurately by extracting emotional features, such as the downward corners of the mouth when somebody expresses sad emotion (Black et al. 2017; Kuusikko et al. 2009).

It has been well‐studied that autistic individuals often experience challenges in forming others' beliefs or intentions (Broekhof et al. 2015; Carpenter et al. 2001; Cattaneo et al. 2007; Rasga et al. 2017; Williams and Happe 2010). One of the most classic paradigms for testing this ability is the *false belief task* (Baron‐Cohen 1989). In this task, participants are presented with a scenario where Sally leaves her apple in the basket and exits the room. Anne, another protagonist, then places the apple in the drawer. The participants would be asked where Sally would look for the apple upon her return. NT children can infer that Sally believes the apple is still in the basket, so they predict she will look there. It demonstrates that NT children form the belief that Sally does not know the apple being moved. In contrast, autistic children form beliefs based solely on their observations: since they have seen the apple placed in the drawer, they expect Sally to look in the drawer. This difference in belief formation between autistic children and NT children highlights the challenges autistic children face in passing the *false belief task*.

Taken together, numerous studies have reported the challenges in each component of ToM processing in autistic individuals. However, behavioural experiments can only help us observe the unique ToM behaviours among autistic individuals, but could not inform which specific component ultimately leads to the differneces in ToM ability.

With the advancement of neuroimaging methods, researchers employ functional Magnetic Resonance Imaging (fMRI) to observe the brain regions involved in the basic cognitive components underlying ToM process. The fMRI enables us to further analyse the neural correlates involved in ToM and address questions that behavioural experiments alone cannot resolve. Next, we will review the neural mechanisms underlying the basic components of the ToM process: environmental perception, information processing, and belief or intention formation.

Firstly, we will delve into the neural correlates of environmental perception. Similar to the behavioural experiments reviewed earlier, we focus here solely on studies adopting visual environmental perception paradigms with fMRI. Several studies have reported that the medial prefrontal cortex (mPFC) may play a key role in attention regulation during environmental perception (Alaerts et al. 2017; Koldewyn et al. 2011). Keehn and his colleagues employed the visual search task and showed that autistic individuals exhibited higher activation in the occipital lobe compared to NT individuals (Keehn et al. 2008), similar to findings in other studies (Lee et al. 2007; Manjaly et al. 2007). Additionally, they also found that autistic individuals showed increased activation in the superior and inferior parietal lobule as well as the superior and inferior frontal gyrus (IFG) than NT individuals (Keehn et al. 2008). The results imply that autistic individuals may increase top‐down control to complete the task. When presented with stimuli containing social information, such as biological motion stimuli, autistic individuals generally exhibit reduced activation in the posterior superior temporal sulcus (STS) compared to NT individuals (Dakin and Frith 2005). In summary, environmental perception typically activates the prefrontal cortex (PFC). When the environment is involved in social information, the posterior STS is recruited for perception. Autistic individuals exhibit diverse activation patterns in these brain regions during environmental perception.

Secondly, regarding the component of information processing, the PFC and subcortical regions play important roles (Yang et al. 2015). Autistic individuals (vs. NT individuals) demonstrate reduced activation in these two brain regions (Adolphs et al. 2001; Bachevalier and Loveland 2006; Zhao et al. 2021). Specifically, in visual spatial working memory tasks, autistic individuals show reduced PFC and cingulate cortex activity compared to NT individuals (Koshino et al. 2005; Luna et al. 2002; Urbain et al. 2015). In verbal working memory tasks, autistic individuals exhibit weaker activation in the frontal lobe, cingulate gyrus, inferior parietal lobe (IPL), and precuneus (PC) compared to NT individuals (Desaunay et al. 2023; Vogan et al. 2018). When it comes to recognition abilities, faces are the most commonly used stimuli. A meta‐analysis on the processing of emotional faces reported reduced hypothalamus activation in autistic individuals compared to their NT counterparts (Aoki et al. 2015). A similar experiment on the emotional judgement task found that autistic individuals exhibit decreased amygdala activation compared to NT individuals (Ishitobi et al. 2011).

Finally, numerous studies have demonstrated that the right temporoparietal junction (TPJ) is a crucial brain region for belief or intention formation (Molenberghs et al. 2016; Vucurovic et al. 2020). Using the cognitive ToM task, Kim and his colleagues found that autistic participants recruited the mPFC, anterior cingulate cortex (ACC), and STS (with its posterior part considered part of the TPJ) (Yang et al. 2015) to a greater extent than NT individuals (Kim et al. 2016). This suggests that autistic individuals need to engage additional PFC resources to compensate for their performance in ToM tasks. Meanwhile, some researchers have attempted to isolate the belief or intention formation stage from ToM tasks (Bardi et al. 2017; Yuk et al. 2018). Employing a modified *false belief task*, Sommer and his colleagues defined the phase where participants observed an agent's actions as the belief formation stage, and found no neural differences between autistic and NT participants in this stage (Sommer et al. 2018). Adopting the Reading the Mind in the Eyes Test (RMET), Holt et al. (2014) discovered that in males, the NT group exhibited enhanced activation in the left IFG, orbitofrontal cortex (OFC), temporal pole (TP), and middle temporal gyrus (MTG) compared to the autistic group. Moreover, in females, the NT group showed greater activation in the left OFC, TP, bilateral IFG, and PFC than the autistic group.

Taken together, within the complex process of ToM, autistic individuals exhibit distinct behavioural and neural activity patterns from NT individuals. However, it remains unclear which specific component or what kind of interaction among components ultimately leads to diverse ToM performances in autistic individuals. Additionally, it is worth noting that most existing studies exploring the neural mechanisms of ToM and its basic components in autistic individuals and their NT counterparts have mainly focused on different activations of certain isolated brain regions. However, since brain regions interact with and influence each other (Shamay‐Tsoory et al. 2010), it is crucial to look at brain networks based on neural connections rather than individual brain regions when discussing brain functions (Mišić and Sporns 2016). Meanwhile, the previous inconsistent findings may be explained by diverse ToM paradigms, emphasising the need for a quantitative meta‐analysis synthesising studies adopting various ToM tasks. Therefore, in the current study, our primary two objectives are to construct a potential neural network for ToM processing in autistic individuals and to identify which specific component(s) may contribute to the differences in ToM performance between autistic and NT individuals. To achieve this goal, we conducted a meta‐analysis to investigate the differences in ToM networks in autistic and NT groups using data from previous fMRI studies. Particularly, we applied a novel seed‐based meta‐approach, Activation Network Mapping (ANM), to examine heterogeneous fMRI findings (Peng et al. 2022). ANM allows us to examine whether heterogeneous fMRI results are located in a set of common networks to improve reproducibility. It makes use of functional networks of independent standardised resting state fMRI connectome from a large sample of healthy participants to identify the flow of activity in processing and team levels.

## 2 Method

### 2.1 Paper Selection

**FIGURE 1:**
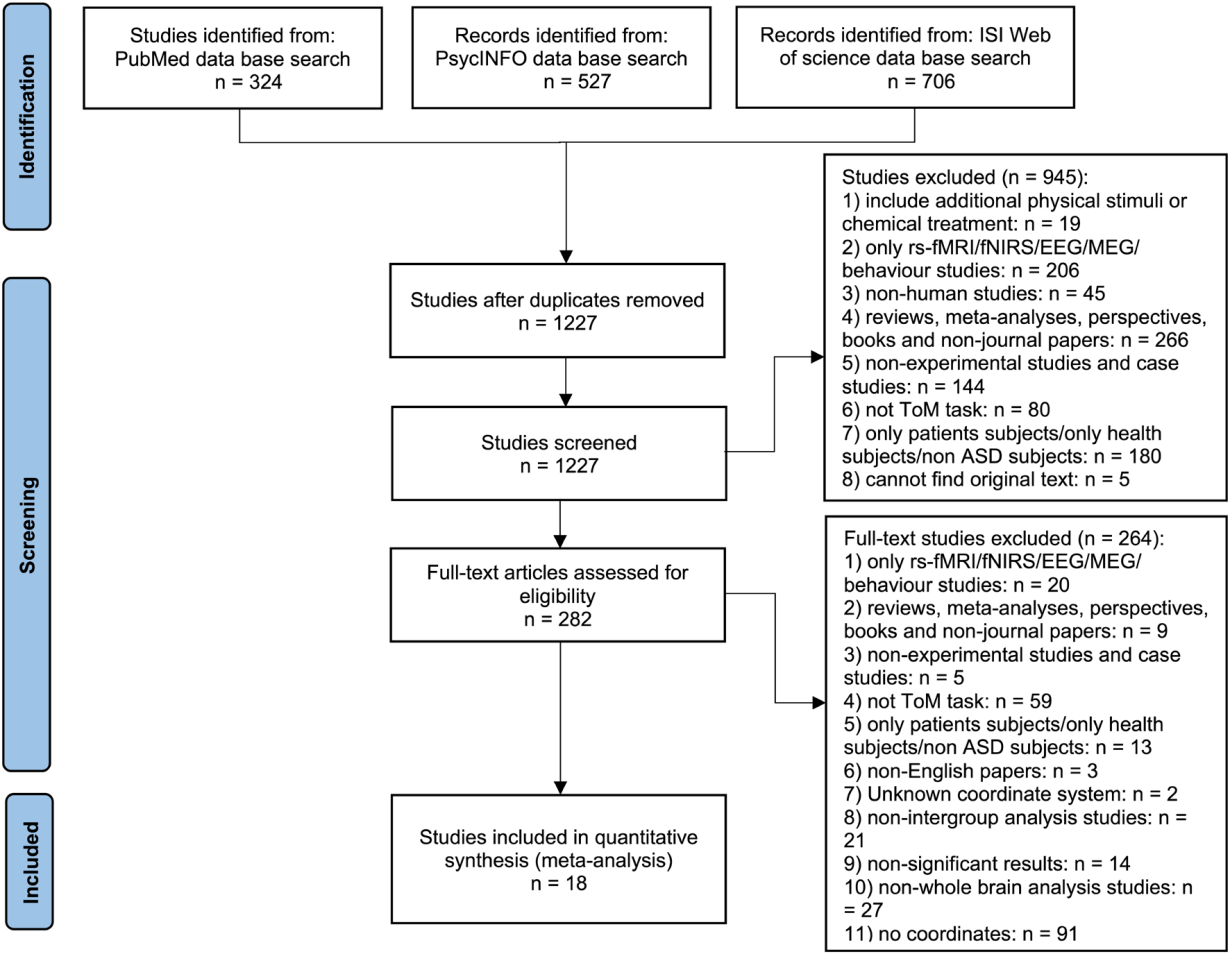
Preferred reporting items for systematic reviews and meta‐analysis (PRISMA) flow diagram for the procedure of study identification.

An initial search of the relevant publications was conducted by a research assistant in July 2024 from the following three databases: PubMed, PsycINFO, and Web of Science. Two researchers independently conducted literature screening. To include as many studies as possible, the search keywords for research methods, Theory of Mind, and autism were as follows: [(fMRI) OR (functional MRI) OR (functional magnetic resonance imaging) OR (neuroimaging)] AND [(Theory of Mind) OR (Theory AND of AND mind) OR (social cognition) OR (social AND cognition) OR (social perception) OR (social AND perception) OR (social behaviour) OR (social AND behaviour) OR (perspective‐taking) OR (perspective AND taking) OR (mentalizing) OR (mind reading) OR (mind AND reading) OR (empathy) OR (empathetic) OR (altruism) OR (sympathy) OR (emotional contagion) OR (compassion)] AND [(autism) OR (PDD‐NOS) OR (pervasive developmental disorder) OR (Asperger)]. Only studies that met the following inclusion criteria were selected: (a) Studies should include ToM tasks‐based fMRI scans and examine ToM ability without additional physical or chemical treatment. (b) Only original research articles should be included. (c) Studies should utilize whole‐brain analyses and intragroup analyses. (d) Studies should recruit a group of autistic participants and a group of NT participants as a control group. (e) Studies should report results in Talairach or MNI coordinates. (f) The full manuscript should be available in English. The process of literature selection followed the guidelines for the Preferred Reporting Items for Systematic Reviews and Meta‐Analyses (PRISMA) (Page et al. 2021). Figure 1 illustrates the process of study search and selection. Finally, 18 studies met all the criteria and were included in the current study. Table 1 shows the abstracts of these selected studies.

**TABLE 1:** Demographic characteristics, tasks and behavioural results of studies included in the meta‐analysis.

Study	*N* (male)		Range: Mean age (SD)		Diagnosis	Task	Performance
	NT	ASD	NT	ASD			
Carter et al. (2012)	13 (11)	12 (9)	7–15: 11.466 (2.63)	8–16: 13.096 (2.39)	Autism diagnoses were based on the ADI‐R, ADOS, and expert clinical opinion. ADOS_COMM: 3.83 (0.94), ADOS_SOC: 7.25 (1.22).	Participants were asked to view “Goofus and Gallant” cartoons and answer which boy was being bad/doing something that he was not supposed to do (social condition) or which picture took place outside (physical condition).	No between‐group differences in error rate or RT.
Dufour et al. (2013)	27 (22)	27 (22)	18–50: 30.6 (9.3)	18–52: 31 (11.5)	Autistic participants received a clinical diagnosis. ADOS_COMM: 3.2 (1.3), ADOS_SOC: 5.9 (2.1).	Participants were presented with verbal stories in English that described a character who acquired a false belief (belief condition) or a physical representation that became false, such as an outdated photograph or map (photo condition).	N/A.
Fan et al. (2014)	21 (21)	24 (24)	—: 19.3 (3.4)	—: 18.4 (2.8)	The diagnosis of autistic participants was confirmed using the DSM‐IV diagnostic criteria and the ADI‐R. ADI‐R_COMM: 15.6 (6.3), ADI‐R_SOC: 19.5 (7.2), ADI‐R_Repeti: 4.9 (2.2).	Participants were asked to view images depicting body parts (hands and feet) being injured accidental or intentionally inflicted by another individual or not were used.	N/A.
Georgescu et al. (2013)	13 (9)	13 (9)	24–26: 30.23 (3)	24–39: 31.23 (4.87)	The autistic participants were diagnosed and recruited in the local autism outpatient clinic. AQ of the autistic group: 38.31 (4.05), AQ of the NT group: 13.85 (3.63).	Participants were asked to view male or female faces with different gaze directions and durations, and to observe and rate the perceived likeability of each face.	No between‐group differences in perceived likeability or RT.
Greene et al. (2011)	22 (19)	22 (20)	10–17: 13.19 (2.44)	9–17: 12.95 (2.46)	The diagnosis of autism participants was confirmed using the ADOS and the ADI‐R, as well as by expert clinical judgement based on DSM‐IV diagnostic criteria. ADOS_COMM: 3.18 (1.84), ADOS_SOC: 7.50 (1.77).	Participants were presented with an eye contact cue or an arrow cue, followed by the appearance of a target object. The target object could appear at the location indicated by the cue or at the opposite location from the cue. The participants were required to report the location of the target object as quickly as possible.	No between‐group differences in error rate or RT.
Greimel et al. (2010)	15 (15)	15 (15)	—: 15 (1.4)	13–17: 14.9 (1.6)	Autistic participants had been diagnosed by experienced clinicians according to ICD‐10 and DSM‐IV. Diagnoses were confirmed by the ADOS‐G and ADI‐R. AQ of the autistic group: 23.8 (8.2), AQ of the NT group: 13.9 (5.8).	Participants were asked to empathise with and judge the emotions displayed by the faces.	The error rate of the autistic group was significantly higher than that of the NT group. There was no difference in RT between the groups.
Hadjikhani et al. (2014)	31 (28)	36 (33)	13–43: 22.5 (7.5)	13–44: 23.5 (8.7)	Experienced clinicians diagnosed autistic participants according to DSM IV‐TR criteria. Diagnoses were confirmed by the ADI‐R and the ADOS. ADOS: 11.1 (4.1), ADI‐R: 42.9 (8.8). AQ of the autistic group: 29.5 (7.5), AQ of the NT group: 13.2 (6.1).	Participants were presented with videos of faces expressing pain or no pain.	N/A.
Kana et al. (2009)	12 (12)	12 (10)	16.6–31.4: 24.4 (3.7)	15.8–35.7: 24.6 (6.9)	The diagnosis of autistic participants was established using the ADI‐R and the ADOS‐G, supplemented with confirmation by expert opinion.	Participants were presented with an animation consisting of a large red triangle and a small blue triangle moving, and were required to choose a word that best described the action after the animation ended.	No between‐group differences in error rate or RT.
Kana et al. (2014)	15 (—)	15 (—)	16–34: 22.28 (1.08)	16–29: 21.14 (0.99)	Autistic participants had received a previous diagnosis based on ADI‐R and ADOS.	The participants were shown a series of comic strips and were required to choose a logical ending to the story. The scenarios depicted required either a physical causal attribution or an intentional causal attribution to complete the task.	The error rate of the autistic group was significantly higher than that of the NT group while making intentional causal attribution. There was no difference in RT between the groups.
Kana et al. (2015)	13 (11)	13 (11)	10–15: 12.7 (1.5)	10–16: 12.6 (1.9)	The diagnosis of autistic participants, as per DSM‐IV guidelines, was established using the ADI‐R and the ADOS‐G, supplemented with confirmation by expert opinion. ADOS_COMM: 4 (0.9), ADOS_SOC: 9.3 (1.6). ADI‐R_word: 24.3 (10), ADI‐R_phrase: 113.3 (278).	Participants were presented with an animation consisting of a large red triangle and a small blue triangle moving (including ToM type, goal‐directed type and random type), and were required to choose a word that best described the action after the animation ended.	The error rate of the autistic group was significantly higher than that of the NT group in ToM condition. There was no difference in RT between the groups.
Kim et al. (2016)	12 (12)	12 (11)	7–18: 11.7 (2.1)	7–18: 12.4 (2.3)	A child and adolescent psychiatrist performed the diagnosis according to the standard DSM‐IV. In addition to clinical diagnosis, the ADI‐R and the ADOS were used to confirm the diagnosis. ADOS_COMM: 4.3 (1.5), ADOS_SOC: 7.8 (3.2).	Participants were presented with a cartoon face (Yoni) and four pictures of the same type of objects, and were asked to infer the picture Yoni was referring to based on the sentence that appeared at the top of the screen and available cues, such as Yoni's gaze, facial expression, etc. This task condition contained both cognitive and affective.	No between‐group differences in error rate or RT.
Libero et al. (2014)	22 (17)	21 (17)	19–36: 24.9 (5.2)	17–40: 25.7 (6.4)	Autistic participants were included if they had a diagnosis of ASD based on the ADI‐R and the ADOS. Diagnoses were verified through patient records retrieved through each participant's clinician.	Participants were shown a series of photos of models interacting with common household objects. In the intention task, participants were asked to determine whether the intention of the model's behaviour was normal; in the means task, participants were asked to judge whether the model's behaviour was normal.	The error rate of the autistic group was significantly higher than that of the NT group in both conditions. There was no difference in RT between the groups.
Redcay et al. (2013)	14 (11)	13 (10)	20–35: 26.64 (5.41)	18–39: 28.46 (6.78)	Autistic participants were diagnosed by an experienced therapist using ADOS. Or participants with a diagnosis on the spectrum as assessed by their personal healthcare providers, the authors also sent the video of their ADOS to their own psychiatrist for final confirmation of the diagnosis. ADOS_COMM: 3.15 (0.95), ADOS_SOC: 6.08 (1.82).	Participants were required to complete a mouse‐catching game. Participants were asked to judge where the mouse might be based on the experimenter's gaze.	No between‐group differences in error rate.
Schneider et al. (2013)	28 (15)	28 (15)	Male: —: 34.27 (9.73) Female: —: 27.85 (7.02)	Male: —: 32.73 (9.97) Female: —: 29.85 (8.02)	Autistic participants were recruited at the local in‐ and outpatient facilities of the Department of Psychiatry, as well as at local self‐ help groups and therapy centers. Inpatients were diagnosed at a special facility for patients with ASD. Patients from self‐help groups and therapy centers were included, if they had received a pre‐diagnosis of ASD by experienced psychiatrists. ADOS‐G of female: 6.83 (3.76), ADOS‐G of male: 7.60 (4.86). AQ of the male autistic group: 34.86 (9.19), AQ of the female autistic group: 41 (4.24), AQ of the male NT group: 11.93 (7.11), AQ of the female NT group: 9.31 (7.92).	Participants were asked to watch video clips of non‐professional actors describing stories related to themselves. These videos included the emotional condition (semantics, rhythm and expression were all emotional) and the neutral condition (semantics, rhythm and expression were all neutral). After the video, participants were asked to give feedback on their own feelings and the feelings of the actors.	The error rate of the autistic group was significantly higher than that of the NT group in both conditions. There was no difference in RT between the groups.
Stroth et al. (2019)	9 (0)	9 (0)	13.9–25: 19.9 (3.6)	12.5–24.5: 18.7 (4.9)	All autistic participants matched the DSM‐IV criteria for ASD and had a confirmed ICD‐10 diagnosis. AQ of the autistic group: 22.6 (10.1), AQ of the NT group: 9.8 (2.5). ADOS_SOC: 7.7 (3.1), ADOS_RRB: 1.0 (0.9), ADOS_COMP: 4.9 (2.1). ADI‐R_COMM: 6.5 (2.7), ADI‐R_SOC: 7.3 (4.5), ADI‐R_STEREO: 2.7 (2.0).	Participants were presented with photos of no pain, physical pain, and social pain conditions depicted from a first‐person perspective. Participants were asked to rate the pain or embarrassment of the social target.	The autistic group rated the targets' embarrassment was significantly higher than that of the NT group. They did not report the RT results.
Sommer et al. (2018)	15 (10)	15 (10)	—: 29.9 (12.2)	—: 28.2 (10.4)	All participants were diagnosed by specialised psychiatrists and psychotherapists according to the ICD‐10 criteria for Asperger syndrome and autism without intellectual disability.	Participants were asked to complete a version of the object transfer false belief task.	No between‐group differences in error rate or RT.
Pelphrey et al. (2005)	9 (8)	10 (9)	15.5–32.4: 23.4 (5.8)	17.9–50.7: 23.2 (9.9)	Diagnoses were based on a history of clinical diagnosis of autism, parental interview (ADI‐R) and proband assessment (ADOS).	The participants were asked to watch a video of a simulated actor. In the video, a cue would appear first, and then the simulated actor's eyes would either look in the direction of the cue (congruent condition) or in the other direction (incongruent condition). The participants were asked to press a button when they saw the eyes move.	No between‐group differences in error rate or RT.
Rosenblau et al. (2016)	22 (16)	20 (14)	19–46: 31.3 (8.5)	19–47: 31.8 (9.3)	All of the participants were diagnosed according to the DSM‐IV criteria for Asperger syndrome and autism without intellectual disabilities. Diagnostic instruments included the ADOS and the ADI‐R. Diagnosis was confirmed using at least one of the gold‐standard instruments ADOS or ADI‐R. Additionally, the diagnosis of Asperger syndrome was confirmed with the Asperger Syndrome and High‐Functioning Autism Diagnostic Interview. ADOS: 10.4 (3.5).	Participants were asked to watch the film in two conditions. In the ToM condition, participants were asked to rely on emotional mentalization or cognitive empathy to infer changes in the protagonist's emotional state. In the body inference condition, participants were asked to judge changes in the protagonist's body movements.	No between‐group differences in error rate or RT.

Abbreviations: ADI‐R, autism diagnostic interview‐revised; ADI‐R_COMM, autism diagnostic interview‐revised communication score; ADI‐R_phase, autism diagnostic interview‐revised phase score; ADI‐R_RRB, autism diagnostic interview‐revised restricted, repetitive, and stereotyped behaviours and interests score; ADI‐R_SOC, autism diagnostic interview‐revised reciprocal social interactions score; ADI‐R_word, autism diagnostic interview‐revised word score; ADOS, autism diagnostic observation schedule; ADOS_COMM, autism diagnostic observation schedule communication score; ADOS_COMP, autism diagnostic observation schedule comparison score; ADOS_SOC, autism diagnostic observation schedule social interaction score; ADOS‐G, autism diagnostic observation schedule‐generic; AQ, autism spectrum quotient; DSM‐IV, diagnostic and statistical manual of mental disorders 4th edition; DSM‐IV‐TR, diagnostic and statistical manual of mental disorders 4th edition text revision; ICD‐10, international statistical classification of diseases and related health problems 10th revision; RT, reaction time; SD, standard deviation.

### 2.2 ANM Analysis

Peak coordinates reported in the selected studies were counted by another researcher, and all Talairach coordinates were converted to MNI coordinates using mni2tal (Lacadie et al. 2008). Firstly, for each peak coordinate, we generated a 6‐mm‐radius sphere centred on it. We used the coactivation function in Neurosynth to combine the spheres from the same study in order to get the experiment‐level inferences. For convenience, we called combined spheres from the same study coactivation seeds. Secondly, we utilized the data from the Brain Genomics Superstruct Project 1000 (GSP1000) Preprocessed Connectome to get the resting‐state functional connectivity (FC) map from coactivation seeds (Cohen et al. 2020). Specifically, GSP1000 Preprocessed Connectome is from the GSP1000 which contains data of a total of 1570 participants (Ages 18–36). However, in the GSP1000 Preprocessed Connectome, 1000 participants (1:1 Male/Female) with high‐quality neuroimaging data were selected and Thomas Yeo's Computational Brain Imaging Group (CBIG) fMRI preprocessing pipeline was adopted for data preprocessing. Thirdly, we used Pearson's correlation coefficient and Fisher's *z* transformation to calculate the Fisher z maps between coactivation seeds and other voxels.

We employed two complementary approaches to acquire group‐level ToM network maps. The first approach is called an overlap map. The Fisher z maps from each study were compared against 0 using the voxelwise one‐sample *t*‐test. Additionally, we set a threshold *t*‐value of 5.66 for the experimental‐level t maps (*p* < 0.05/285,903 voxels = 1.76 × 10^−7^). Afterwards, these experimental‐level t maps were binarized. Eventually, in order to obtain the overlap map, we overlapped the experimental‐level t maps and brain regions were retained in the network map only if they had functional connections with more than 60% of the coactivation seeds.

In addition to the overlap map, the second approach is the t map. We averaged 1000 participant‐level Fisher z maps in each experiment to engender an experiment‐level mean Fisher z map. Furthermore, to get the t map, the experiment‐level mean Fisher z maps were compared against 0 using a voxelwise one‐sample *t*‐test thresholding *t*‐value of 5.66 (*p* < 0.05/285,903 voxels = 1.76 × 10^−7^). Finally, the t map showed brain regions which were significantly connected to coactivation seeds.

According to the above mentioned procedure, we generated the overlap map and t map for both the autistic group and the NT group. Moreover, we contrasted the autistic group with the NT group for both the overlap map and the t map.

## 3 Results

This study comprised 18 individual studies, each with a single experiment, totally involving 314 NT participants and 328 autistic participants.

### 3.1 Behavioural Results

Table 1 summarised the behavioural findings from all studies included in the meta‐analysis. Autistic individuals and their NT counterparts exhibited similar levels of error rate and response times in the ToM tasks. Specifically, excluding two studies that either did not report or were not concerned with behavioural outcomes, only 37.5% of the studies (6/16) reported a higher error rate in autistic individuals than that in their NT peers. The remaining 10 studies found that autistic individuals could complete the ToM task just as well as NT individuals, with no significant difference in error rates between the two groups. Additionally, none of the included experiments found a significant difference in response times between the two groups.

### 3.2 ToM Network in the NT Group

**FIGURE 2:**
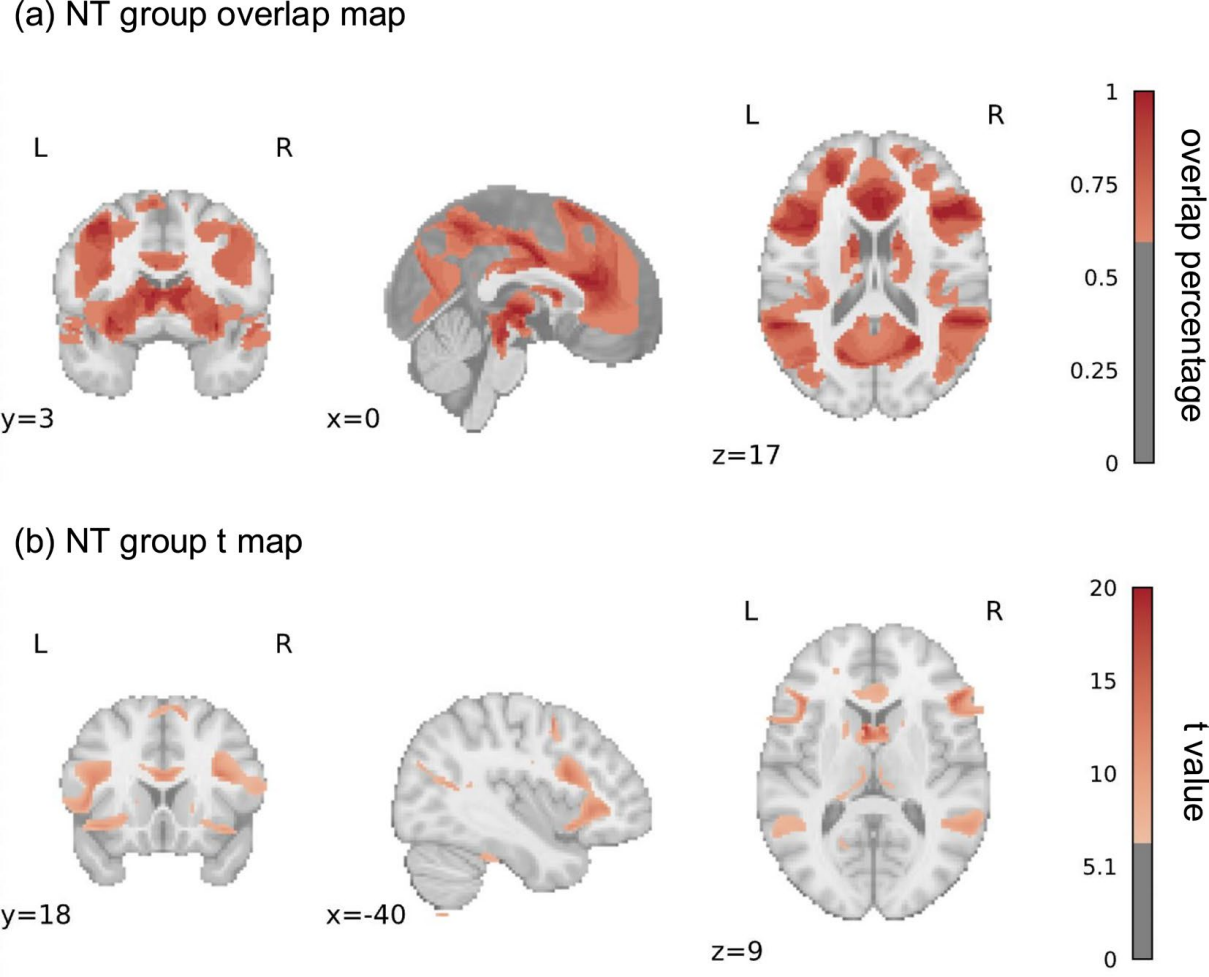
The overlap map and t map of the NT group.

The results were summarised in Table 2. The results were presented in descending order of the overlap ratio. Firstly, the overlap map revealed that the 100% coactivation seeds were functionally connected to the left thalamus in the NT population (See Figure 2). Secondly, the other brain regions of the limbic system, like the left‐caudate, were functionally connected to over 60% (11/18) of coactivation seeds. Lastly, the parietal lobe regions, such as the angular gyrus and lingual gyrus, were substantially connected to coactivation seeds in the NT group during the different ToM tasks. As for the t map, cingulum and caudate showed strong FC to coactivation seeds. Several cortex areas, such as OFC, PC, and IFG, also contributed to the NT group's ToM network. The similarity (Person correlation) between the overlap map and the t map of the NT group was 0.65.

**TABLE 2:** Peaks of the autistic group's ToM network.

Map type	MNI coordinates			AAL label	BA label
	*x*	*y*	*z*		
Overlap map	−8	−26	2	Thalamus_L	Left‐Thalamus (50)
	−8	−50	48	Precuneus_L	Left‐BA7
	52	−42	20	Temporal_Sup_R	Right‐BA22
	−4	6	10	Caudate_L	Left‐Caudate (48)
	16	−78	−60	Cerebelum_8_R	Right‐BA19
	−14	−80	−58	Cerebelum_7b_L	Left‐VisualAssoc (18)
	58	−58	−48	Cerebelum_Crus2_R	Right‐BA20
	−18	−70	−44	Cerebelum_8_L	Left‐BA19
	−8	−30	26	Cingulum_Post_L	Left‐BA23
	−62	−26	4	Temporal_Mid_L	Left‐BA22
	−26	36	−8	Frontal_Inf_Orb_L	Left‐BA47
	−58	−2	−4	Temporal_Sup_L	Left‐BA22
	−60	−16	0	Temporal_Mid_L	Left‐BA22
	−60	−12	0	Temporal_Mid_L	Left‐BA22
	18	−26	60	Precentral_R	Right‐PrimMotor (4)
	20	−24	64	Precentral_R	Right‐PrimMotor (4)
	20	48	28	Frontal_Sup_R	Right‐BA9
	20	50	24	Frontal_Sup_R	Right‐BA10
	−62	−22	4	Temporal_Sup_L	Left‐BA22
*t* map	−4	10	10	Caudate_L	Left‐Caudate (48)
	4	8	8	Caudate_R	Right‐Caudate (48)
	−20	−38	32	Cingulum_Mid_L	Left‐BA23
	14	−82	−54	Cerebelum_7b_R	Right‐VisualAssoc (18)
	58	−58	−46	Cerebelum_Crus2_R	Right‐Fusiform (37)
	26	−40	30	Angular_R	Right‐PrimSensory (1)
	10	−28	2	Thalamus_R	Right‐Thalamus (50)
	−10	−48	48	Precuneus_L	Left‐BA7
	−12	−30	2	Thalamus_L	Left‐Thalamus (50)
	34	20	20	Frontal_Inf_Tri_R	Right‐BA44
	−20	−44	40	Cingulum_Mid_L	Left‐BA31
	−38	−60	16	Occipital_Mid_L	Left‐BA39
	−2	4	−38	Fusiform_L	Left‐Parahip (36)
	20	−44	42	Precuneus_R	Right‐BA31
	−44	26	14	Frontal_Inf_Tri_L	Left‐BA45
	46	−50	12	Temporal_Mid_R	Right‐Fusiform (37)
	−16	−44	42	Precuneus_L	Left‐BA31

### 3.3 ToM Network in the Autistic Group

**FIGURE 3:**
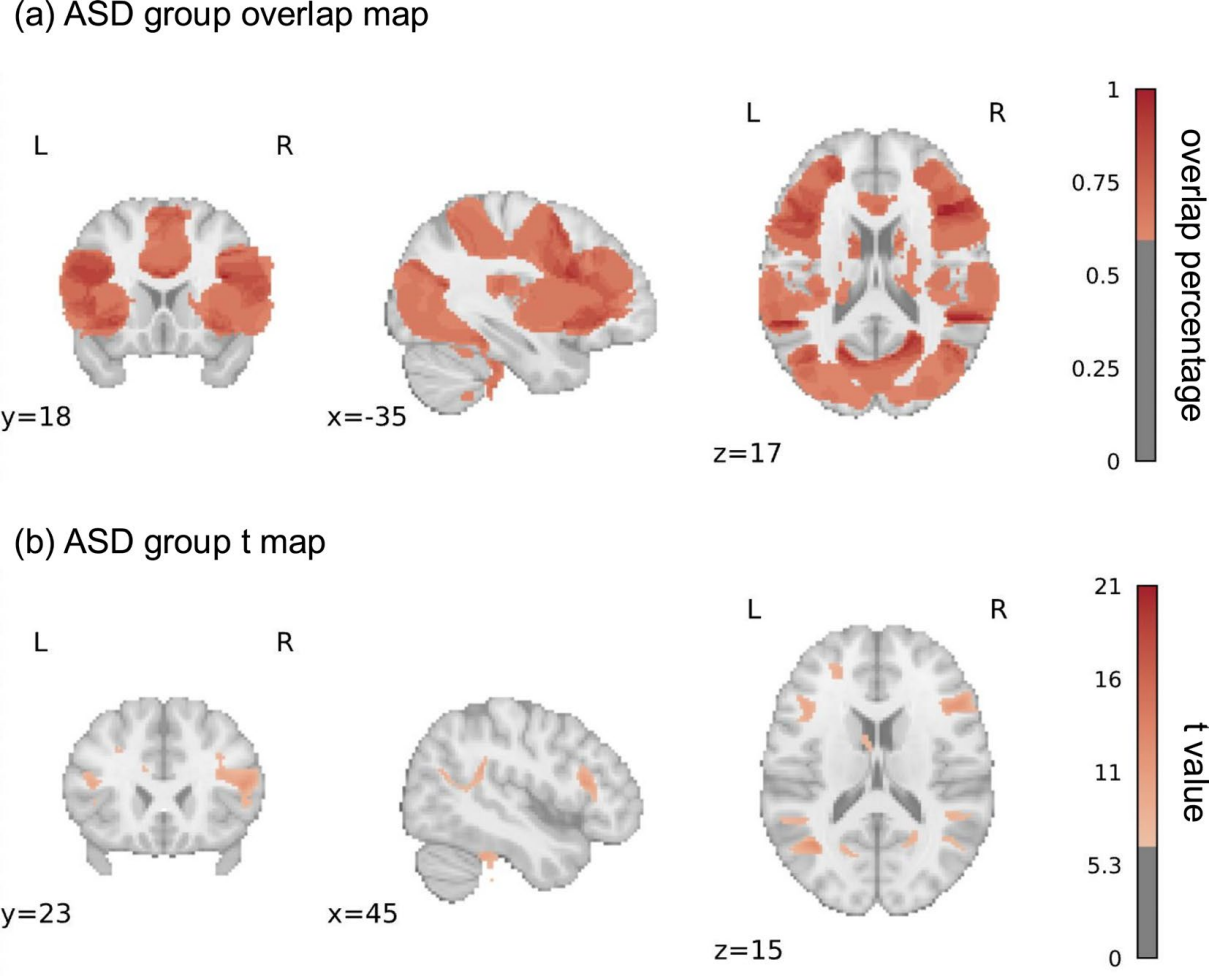
The overlap map and t map of the autistic group.

The results were summarised in Table 3 and illustrated in Figure 3. The results were presented in descending order of the overlap ratio. Firstly, the overlap map revealed that the left thalamus, left PC and right superior temporal lobe robustly plugged into the ToM network of the autistic population. That is, these brain regions were functionally connected to 100% coactivation seeds. Secondly, the frontal brain regions, such as OFC, precentral and paracentral gyrus, of autistic participants were functionally connected with over 60% (11/18) coactivation seeds. In addition, subcortical regions including the hippocampus and cingulum were also functionally connected with over 60% (11/18) coactivation seeds. The t map showed significant results in subcortical areas. The largest clusters were found in the caudate and cingulum. Similar to the overlap map, the t map also revealed the same ToM network pattern in the thalamus and hippocampus. The similarity between the overlap map and the t map (Person correlation) of the autistic group was 0.45.

**TABLE 3:** Peaks of NT groups's ToM network.

Map type	MNI coordinates			AAL label	BA label
	*x*	*y*	*z*		
Overlap map	−4	−12	0	Thalamus_L	Left‐Thalamus (50)
	−6	6	12	Caudate_L	Left‐Caudate (48)
	0	−4	32	Cingulum_Mid_R	Right‐BA24
	−42	18	−10	Frontal_Inf_Orb_L	Left‐BA47
	50	−38	−36	Cerebelum_Crus2_R	Right‐BA20
	−38	−60	42	Angular_L	Left‐BA39
	18	−22	20	Caudate_R	Right‐Caudate (48)
	−30	−74	46	Parietal_Inf_L	Left‐BA39
	−30	−76	44	Parietal_Inf_L	Left‐BA39
	−16	−60	40	Precuneus_L	Left‐BA7
	−26	−40	−18	Fusiform_L	Left‐Fusiform (37)
	−16	−22	20	Thalamus_L	Left‐Caudate (48)
	−18	−60	2	Lingual_L	Left‐VisualAssoc (18)
	−22	−62	44	Parietal_Sup_L	Left‐BA7
	−30	−72	48	Parietal_Inf_L	Left‐BA39
	−22	−64	46	Parietal_Sup_L	Left‐BA7
	−32	−68	48	Parietal_Inf_L	Left‐BA7
	−32	−70	36	Occipital_Mid_L	Left‐BA39
	−58	−50	0	Temporal_Mid_L	Left‐Fusiform (37)
	−22	−66	−6	Lingual_L	Left‐BA19
	46	−54	44	Parietal_Inf_R	Right‐BA39
*t* map	−16	−34	30	Cingulum_Mid_L	Left‐BA23
	58	−58	−46	Cerebelum_Crus2_R	Right‐Fusiform (37)
	−4	10	10	Caudate_L	Left‐Caudate (48)
	38	34	−6	Frontal_Inf_Orb_R	Right‐BA47
	52	−48	12	Temporal_Mid_R	Right‐BA39
	−34	10	−10	Insula_L	Left‐Insula (13)
	−10	10	62	Supp_Motor_Area_L	Left‐BA6
	18	−34	30	Cingulum_Mid_R	Right‐BA23
	−14	−48	46	Precuneus_L	Left‐BA7
	12	−28	6	Thalamus_R	Right‐Thalamus (50)
	2	24	18	Cingulum_Ant_R	Right‐BA24
	−36	4	52	Frontal_Mid_L	Left‐BA6
	−54	−46	20	Temporal_Sup_L	Left‐BA39
	−14	−38	38	Cingulum_Mid_L	Left‐BA31
	20	−64	24	Precuneus_R	Right‐BA31

### 3.4 Contrast Between the Autistic and the NT Group

**FIGURE 4:**
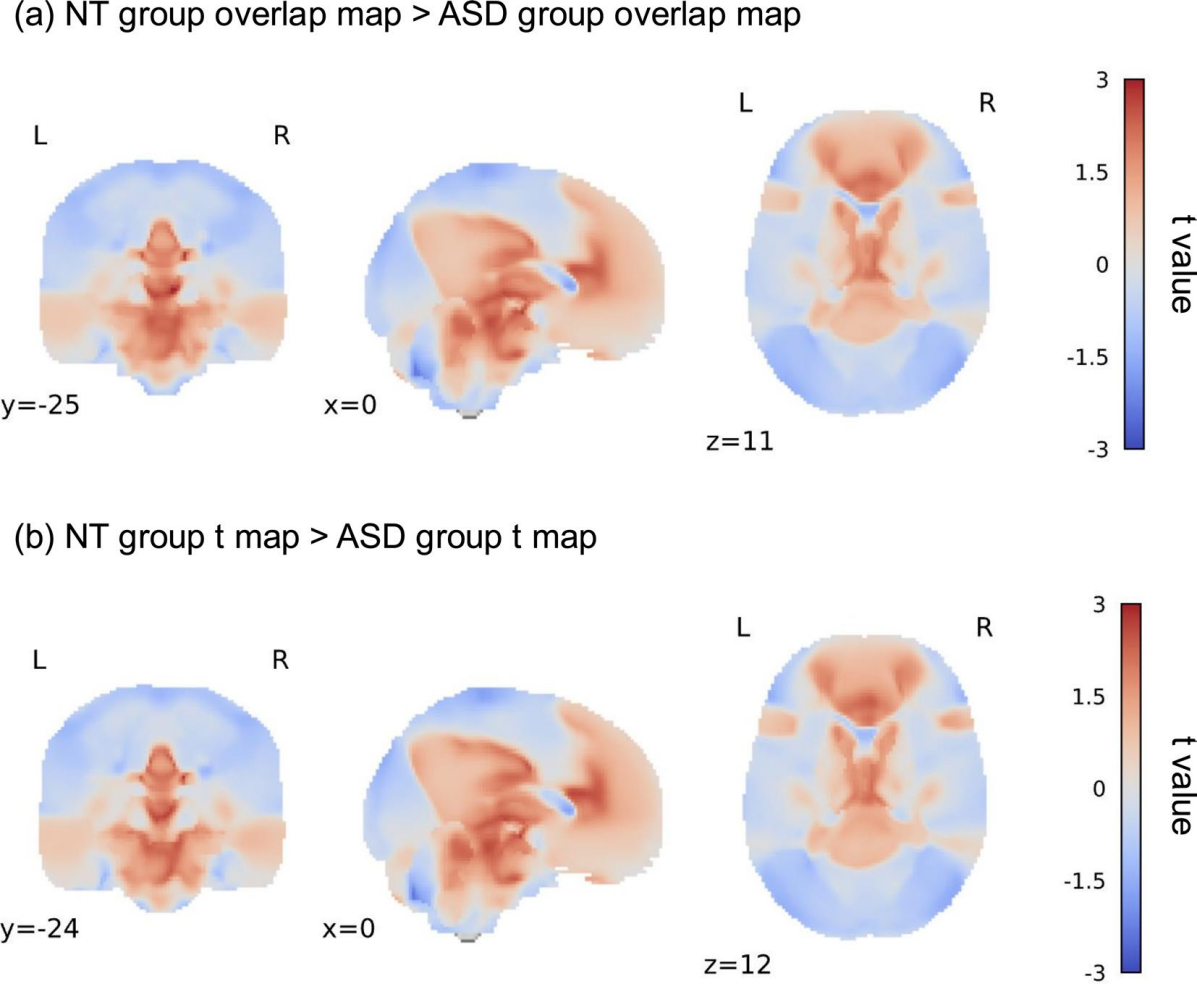
The comparison result of the overlap map and the t map.

We compared the autistic group and the NT group on both the overlap map and the t map. Table 4 and Figure 4 illustrated the results. The contrast results of the overlap map revealed that the limbic system, like the right thalamus, was more likely to participate in the ToM network of the NT group as compared to that of the autistic group. In the meantime, several cortex areas, such as the inferior temporal lobe (ITL) and left IPL, in the NT group's overlap map were also more connected with coactivation seeds than those in the autistic group's overlap map. The group contrast of the t map showed a similar result. Results revealed that the limbic system was more likely to be included in the t map of the NT group's ToM network than that of the autistic group's ToM network. Moreover, the superior frontal lobe and left‐fusiform were more likely to contribute to the ToM network in the NT group's t map than in the autistic group's t map. In addition, for both the overlap map and t map, the group contrast results revealed that the right cerebellum in the NT population was more likely to be associated with coactivation seeds than in the autistic population when performing ToM tasks.

**TABLE 4:** Peaks of compare NT ToM network with autistic ToM network.

Map type	MNI coordinates			AAL label	BA label
	*x*	*y*	*z*		
Overlap map	8	−26	6	Thalamus_R	Right‐Thalamus (50)
	2	−20	4	Thalamus_R	Right‐Thalamus (50)
	14	−28	26	Cingulum_Mid_R	Right‐BA23
	14	−16	28	Caudate_R	Right‐BA23
	44	−54	−64	Cerebelum_8_R	Right‐BA20
	34	−54	−66	Cerebelum_8_R	Right‐BA20
	40	−10	−54	Temporal_Inf_R	Right‐BA20
	28	−10	−54	Fusiform_R	Right‐BA20
	42	−52	−64	Cerebelum_8_R	Right‐BA20
	52	−52	−58	Cerebelum_7b_R	Right‐BA20
	−44	−14	−52	Temporal_Inf_L	Left‐BA20
	−50	−14	−50	Temporal_Inf_L	Left‐BA20
	−24	−32	36	Parietal_Inf_L	Left‐PrimSensory (1)
	14	76	−10	Frontal_Med_Orb_R	Right‐BA10
	62	−20	−42	Temporal_Inf_R	Right‐BA20
*t* map	8	−26	6	Thalamus_R	Right‐Thalamus (50)
	2	−20	4	Thalamus_R	Right‐Thalamus (50)
	14	−24	28	Cingulum_Mid_R	Right‐BA23
	−12	−14	30	Cingulum_Mid_L	Left‐BA23
	44	−54	−64	Cerebelum_8_R	Right‐BA20
	34	−54	−66	Cerebelum_8_R	Right‐BA20
	42	−60	−64	Cerebelum_8_R	Right‐BA20
	42	−52	−64	Cerebelum_8_R	Right‐BA20
	32	−70	−64	Cerebelum_8_R	Right‐Fusiform (37)
	−12	−96	−2	Calcarine_L	Left‐VisualAssoc (18)
	52	−52	−58	Cerebelum_7b_R	Right‐BA20
	−32	10	74	Frontal_Sup_L	Left‐BA6
	−48	6	64	Precentral_L	Left‐BA6
	42	−6	24	Rolandic_Oper_R	Right‐BA6
	−30	12	74	Frontal_Sup_L	Left‐BA6
	−30	−2	−54	Fusiform_L	Left‐BA38

## 4 Discussion

As previously reviewed, the process of ToM involves multiple components. However, prior research has yet to elucidate the neural mechanisms underlying ToM in autistic individuals or identify which specific components contribute to the differences in ToM abilities between autistic and NT individuals.

### 4.1 Neural Basis of Typical ToM Processing

To address previous research gaps related to the underlying mechanisms of ToM processing, our first objective is to synthesise previous findings utilising fMRI and exploring the neural activation in NT and autistic populations during ToM tasks. This synthesis will help identify the brain network involved in ToM processing in both groups and evaluate whether the implicated regions align with our hypothesised basic components underlying the ToM process. Specifically, by synthesising coordinate‐based activation data from 18 ToM studies, our findings revealed similar significant involvement of the following regions across various ToM tasks in both NT and autistic individuals: the left middle occipital gyrus (l‐MOG), left TPJ (l‐TPJ), right TPJ (r‐TPJ), PC, left‐insula, STS, superior frontal cortex (SFC) and medial frontal cortex (MFC).

In the introduction, we initially proposed that the basic components underlying the ToM process can be divided into three components: environmental perception, information processing, and belief or intention formation. Next, we will discuss our results in light of previous research on the functions of the brain regions and their relationship with behaviour that shows cross‐population similarities in our findings and attempt to map these results onto the basic components framework we have proposed.

As stated in the introduction, the first component involved in ToM is the perception of surrounding visual stimuli. The l‐MOG has been identified as a critical component of the visual cognitive network, playing an essential role in the perception of visual stimuli and movement (Geng et al. 2022; Lan et al. 2024). In line with this, we hypothesise that the activation of l‐MOG observed in both NT and autistic populations within the ToM network reflects its involvement in environmental perception, consistent with the proposed model.

Next, we proposed that after perceiving visual stimuli, processing the embedded social information is essential for completing subsequent ToM processes. Our analysis identified the PC and insula as integral brain regions of the ToM network. The PC is involved in visuospatial and motor imagery (Dadario and Sughrue 2023) and plays a key role in transmitting decoded information to the motor and visual cortices (Jitsuishi and Yamaguchi 2021). It is also central to several important brain networks, including the default mode network (DMN) and central executive network (CEN) (Cavanna and Trimble 2006). This suggests that different regions of the PC are interconnected with distinct parts of these networks to decode and transmit information, supporting complex cognitive functions such as self‐referential processing, memory, and emotional regulation. Similarly, the insula, especially the anterior insula, processes and integrates sensory information from both internal and external sources (Sterzer and Kleinschmidt 2010). Specifically, the insula is thought to be the primary region responsible for processing interoceptive information (Bamiou et al. 2003; Ibanez et al. 2010; Naqvi et al. 2007), which is a function crucial for emotional experiences (Couto et al. 2013). Like the PC, the insula exhibits increased activation in tasks involving perception, intentional behaviour, and consciousness (Gasquoine 2014; Wang et al. 2019). Additionally, both the PC and the insula are involved in self‐attributional processes (Cabanis et al. 2013). Thus, we hypothesise that the activation of the PC and insula within the ToM network of both autistic and NT individuals reflects their roles in information processing and integration, consistent with our model, where these regions correspond to the second component of the basic cognitive framework.

In our proposed model, the final stage of the ToM process involves belief or intention formation. Our findings align with this model, showing the activation of several brain regions associated with ToM in both autistic and NT population, including the TPJ (right STS/left IPL) and the middle and superior frontal lobes. Much research has explored the roles of the TPJ, MFC, especially the mPFC, and SFC in ToM (Frith and Frith 2012; Schurz et al. 2014; Van Overwalle 2009). The SFC is uniquely situated at the crossroads of key brain networks, including the DMN, the dorsal attention network, and the CEN (Schaefer et al. 2018; Yeo et al. 2011). This positioning suggests that the SFC plays a crucial role in integrating and coordinating higher‐order cognitive functions, such as attention allocation and cognitive control (Engen and Anderson 2018; Focquaert and Platek 2006). Moreover, the left superior frontal gyrus has been implicated in self‐awareness processes (Goldberg et al. 2006; Lee et al. 2017). The mPFC is central to mental state attribution, both for oneself and others, particularly during action planning and execution (Denny et al. 2012; Frith and Frith 2006; Ninomiya et al. 2018). Meta‐analyses have further broken down the specific roles of these regions: the posterior zone is linked to monitoring movement and performance, the middle zone to cognitive control, pain, and affect, and the anterior zone to reward processing, social cognition, and episodic memory (de la Vega et al. 2016). The TPJ, in particular, is essential for understanding the intentions of others and engaging in perspective‐taking. As initially proposed by Saxe (2006), it is a core region in processing information related to the mental states of others. Additionally, a meta‐analysis found significant TPJ activation during implicit ToM processes, particularly in belief formation (Boccadoro et al. 2019). Together, these regions interact to enable individuals to process, integrate, and synthesise social information, facilitating more sophisticated cognitive functions. These brain regions allow us to form interpretations and predictions about the thoughts and behaviours of both us and others, representing the final, crucial component of the ToM cognitive model we have proposed.

### 4.2 Functional Significance of Altered Activation Patterns in Autistic Individuals

Our second research objective is to compare brain network differences between the NT and autistic individuals during ToM tasks, aiming to uncover the underlying reasons for the observed disparities in ToM performance. Our analysis revealed that, compared to NT individuals, autistic individuals showed less significant FC with coactivation seeds in brain regions such as the superior temporal lobe, IPL, right thalamus, right caudate, and ITL. In the following section, we will interpret these findings within the framework of the ToM cognitive model we proposed, discussing the identified brain regions and their associated functions.

Our study revealed that autistic individuals exhibited a reduced activation probability in the right thalamus, cingulate cortex, and caudate nucleus compared to NT individuals when performing ToM tasks. These brain regions are critical for the limbic system and are closely involved in processing social behaviours and emotions. The caudate, as a part of the striatum, is sensitive to goal‐oriented behaviours (Graff‐Radford et al. 2017). ToM requires the assessment of the agent's target, and thus the weakening of caudate activation affects the performance in ToM tasks (Votinov et al. 2021). The thalamus and caudate are also essential for processing sensory inputs and facilitating communication between subcortical and cortical areas (Sherman and Guillery 2002; Simpson et al. 2010). Additionally, the cingulate cortex and caudate nucleus contribute to attributing emotional significance to stimuli and supporting joint attention, which is vital for distinguishing one's own mental states from others and inferring others' intentions (Barbas 2007; Williams et al. 2005). Joint attention, involving mirror self‐recognition or visual perspective‐taking, is essential for individuals to differentiate their own mental states from those of others and to infer others' intentions (Sodian and Kristen‐Antonow 2015). The reduced activation in these regions in autistic individuals aligns with the information processing component of our proposed ToM model.

Previous research has found differences in sensory processing and integration of social information between autistic individuals and NT individuals (Rudie et al. 2012). These sensory‐perceptual differences in autistic individuals have been associated with disruptions in local thalamic connectivity and thalamocortical networks (Hardan et al. 2008; Tomasi and Volkow 2019). Additionally, research indicates a negative correlation between the strength of connections among the cingulate, caudate, and various cortical regions, including the limbic lobe and medial frontal gyrus, and the severity of the autistic condition (Hoffmann et al. 2016). Since many brain areas in the cortex are responsible for processing complex social signals, this long‐distance underconnectivity, which has also been identified, may lead to unique ToM function in the autistic population due to the decreased of efficient signal transmission between subcortical and intercortical areas (Williams 2011).

Additionally, it is worth noting that previous studies have revealed that ToM is a cognitive process that requires bilateral brain coordination (Santiesteban et al. 2012; Saxe and Kanwisher 2003). In particular, the right hemisphere plays an indispensable role in the normal formation of ToM (Brownell et al. 2000; Mitchell 2008; Shamay‐Tsoory et al. 2005). Moreover, it has been found that autistic individuals exhibit different lateralisation in various brain functions (Li et al. 2023). Therefore, the inability to effectively engage both the left and right brain regions associated with information processing and coordination during ToM tasks may contribute to the behavioural differences observed between autistic and NT individuals. Specifically, autistic individuals have more difficulty accurately judging the agent's intentions in ToM tasks than NT individuals.

Secondly, the right cerebellum showed weaker activation in the ToM brain network of autistic individuals compared to NT individuals. Traditionally linked to motor coordination, the cerebellum is also involved in cognitive and emotional processing (Stoodley 2012). For example, one study comparing autism with cerebellar degenerative diseases has shown that the posterior right cerebellum is key to emotional reasoning and facial expression recognition, whereas the middle cerebellum is linked to cognitive reasoning difficulties. Damage to this region can impair understanding of others' beliefs and intentions in tasks involving belief comprehension and reasoning (Clausi et al. 2021), thereby affecting the accuracy of autistic individuals in ToM tasks. Additionally, resting‐state FC studies have shown reduced cerebellar connectivity with the prefrontal, parietal, and temporal lobes in autism (Khan et al. 2015). In summary, our findings align with previous research, suggesting that reduced cerebellar activation in autism reflects its role in ToM tasks, and may indicate different cerebellar recruitment in autistic individuals compared to NT individuals.

Thirdly, our findings initially indicated that in the ToM brain network of autistic individuals, the bilateral ITL, right OFC, and left IPL showed weaker activation compared to the network of NT individuals when performing the task.

The TPJ, includes both the IPL and the caudal location of the STS (also called pSTS). The pSTS, an integral component of the social brain, plays a significant role in processing social perception, ranging from basic recognition of biological movements like body gestures and eye movements to more intricate social cognitive processes (Brothers 1990; Dahl et al. 2010; Thompson and Parasuraman 2012). Specifically, in the domain of brain function related to ToM, it has been identified as a core of processing information associated with the mental states of others initially proposed by Saxe (2006). Thus, the current finding reconfirmed the crucial function of the TPJ in facilitating the detecting and describing of the mental states of others in ToM tasks, whether inferring emotions or joint attention. Whilst both the IPL and pSTS in the TPJ, are responsible for the representation of mental states as the initial stage of ToM (Schaafsma et al. 2015; Schurz et al. 2021; Stacy et al. 2024; Yang et al. 2015). The OFC is also a crucial brain region of the so‐called “social brain” (Brothers 2002; Carrington and Bailey 2009). Abnormal functioning in this region can impair an individual's ability to consider social norms, moral principles, and rules during social cognition, resulting in difficulties in adjusting behaviour in response to socially aversive cues during ToM tasks (Beer et al. 2006; Krajbich et al. 2009; Kringelbach and Rolls 2004). In summary, these three brain regions correspond to the belief formation component of ToM in our model. The differences in activation of these regions in the ToM network between autistic individuals and NT individuals may explain the behavioural differences observed in ToM tasks.

In summary, the thalamus, caudate, and cingulum play crucial roles as neural components involved in information processing and transmission. These structures facilitate efficient collaboration between different brain regions, aiding individuals in accurately representing and processing both their own thoughts and emotions and those of others (Preckel et al. 2018). Therefore, differences in ToM task performance between autistic and NT individuals may also stem from divergent roles of the thalamus, caudate, and cingulum in these tasks, hindering effective processing and reasoning of gathered information, thereby affecting accurate inference of others' intentions. Additionally, based on our findings, the engagement of the TPJ and OFC within the ToM network varies significantly more among individuals on the autism spectrum compared to NT individuals. The TPJ, involved in perspective‐taking and empathy, and the OFC, essential for evaluating social cues and adjusting behaviour based on social context, show less consistent activation in autistic individuals. This variability potentially contributes to differences in their ToM abilities. Reduced involvement of the superior temporal lobe in ToM tasks among autistic individuals may further impair their ability to establish an understanding of others' intentions and take appropriate actions, leading to distinct performance patterns in these tasks.

This study employed an innovative methodology that integrated overlap maps and t maps from existing literature to construct comprehensive ToM network maps for both autistic and NT individuals. Instead of directly calculating the probability of FC within specific brain regions for each group, we compared these resulting maps to identify key differences. Although we did not perform explicit FC calculations for the autistic group, this approach allows us to draw a meaningful network regarding how differences in brain region connectivity contribute to the divergent ToM performance observed in the autistic and NT individuals. Our methodology provides valuable insights into the neural network of ToM, emphasising specific FC patterns in the autistic population that may underlie their distinct cognitive profile. Furthermore, these findings allow us to infer which potential components may relate to the unique ToM performance characteristics observed in autistic individuals, thereby advancing our understanding of the neurobiological basis of social cognition differences in autistic population.

### 4.3 Limitations and Future Directions

When analysing the findings of this research, it is imperative to take into account a number of constraints. Firstly, the number of valid coordinates included in this study is constrained by the availability of pertinent research. This is because acquiring fMRI data from autistic individuals is challenging. The sensory discomfort caused by fMRI leads to intolerable motion artefacts in some participants' data. Thus the researchers have to exclude these data from analysis. It is hoped that forthcoming research can explore the development of more autism‐friendly fMRI experimental environments, and deliberately concentrate on the neural regions and functional connectivity elicited by the ToM task among autistic individuals. This will promote a deeper understanding of neurodiverse populations including autistic individuals. Furthermore, it should be noted that the present study did not incorporate medication and co‐morbidities of the participants as moderating variables in the analysis. This is due to the fact that the studies included in the analysis barely accounted for these characteristics of the participants. However, previous studies have suggested that co‐morbidities may influence the altered brain activity of participants while completing ToM tasks (Ilzarbe et al. 2020). There is a desire for further research to study the influence of co‐occurring medical conditions and co‐morbidities in autistic individuals on their ToM‐related neural activities. Third, this study considered all autism syndromes as a continuum spectrum and did not classify them. Due to the intricate classification of autism as a spectrum disorder, further investigations will be required to accurately classify autistic individuals.

The findings of this study carry several clinical and translational implications. First, autistic individuals have encountered challenges in both diagnosis and subsequent intervention (Hus and Segal 2021). Neuroimaging approaches offer a promising avenue for enhancing diagnostic accuracy and assessment. This meta‐analysis, which integrated data from 328 autistic individuals and 314 NT counterparts, and employed the novel analytical method to improve reproducibility, identified candidate brain regions that may exhibit differential activation during ToM tasks. These brain areas warrant further investigation as potential biomarkers for the autistic condition, particularly in their specificity for assessing social information, thereby supporting early diagnosis and prognostic evaluation. Second, the study sheds light on possible mechanisms underlying the distinctive social behaviours observed in autistic individuals during ToM tasks. Although various ToM‐based interventions, such as Thought Bubble Training (Wellman et al. 2002), have been developed, evidence for their long‐term efficacy remains limited (Fletcher‐Watson et al. 2014). Our proposed model advocates for deconstructing ToM into three components and targeting these individually through intervention. Future research should explore information‐processing‐based strategies to support autistic individuals.

## 5 Conclusion

The present meta‐analysis provides a comprehensive overview of the brain networks that are likely to appear during ToM tasks. Our findings confirmed the important role of these regions of the l‐TPJ/r‐TPJ, PC, STS and mPFC, thalamus, and caudate‐limbic system in the execution of ToM function in individuals. Furthermore, the current study revealed that ToM neural network of autistic individuals was less likely to involve the STS, right thalamus, right caudate, and ITL in ToM tasks, and a weaker FC in the right thalamus, right caudate, and inferior temporal lobe with coactivation seeds in the ToM network of autistic group, as evidenced by the t map and overlap map analyses. These findings suggest this unique ToM network and different functional connections between the aforementioned regions may lead to challenges in processing and integrating information in autistic individuals. This may underlie the distinct ToM function observed in the autistic group relative to the NT group. In general, the outcomes of this investigation provide insight into the neural underpinnings of ToM and carry significant ramifications for the implementation of ToM‐based interventions in autistic children.

## Ethics Statement

The authors have nothing to report.

## Conflicts of Interest

The authors declare no conflicts of interest.

## Data Availability

The data that support the findings of this study are available from the corresponding author upon reasonable request.
